# Climate change and future challenges to the sustainable management of the Iraqi marshlands

**DOI:** 10.1007/s10661-023-12168-8

**Published:** 2023-12-13

**Authors:** Ala Hassan Nama, Imzahim A. Alwan, Quoc Bao Pham

**Affiliations:** 1 Department of Water Resources Engineering, University of Baghdad, Baghdad, Iraq; 2 Civil Engineering Department, University of Technology, Baghdad, 10066 Iraq; 3 Faculty of Natural Sciences, Institute of Earth Sciences, University of Silesia in Katowice, Będzińska Street 60, 41-200 Sosnowiec, Poland

**Keywords:** Iraqi marshlands, Restoration plans, Climate change, Inundation rates, Sustainability of ecosystem

## Abstract

The application of restoration plans for the Iraqi marshlands is encountering significant challenges due to water scarcity and the impacts of climate change. This paper assesses the impact of water scarcity on the possibility of continuing the application of restoration and sustainable management plans for the main marshlands in Iraq. This assessment was conducted based on the available data and expected situation of available water resources under climate change conditions until the year 2035. Additionally, a satellite image–based index model was prepared and applied for the period 2009–2020 to obtain the spatiotemporal distribution of the restored marshlands. The results show that the shortage in water resources and insufficient inundation rates prevented the adequate application of the restoration plans. Also, applying the scenarios of distributing the deficit equally over all water demand sectors (S1) and according to the percentage of demand for each sector (S2) shows that the expected deficit in available water for the three marshes by the years 2025 and 2035 will be approximately 25% and 32% for S1 and 9% for S2. Consequently, the considered marshes are expected to lose approximately 20 to 33% of their eligible restoration areas. Accordingly, looking for suitable alternatives to support the water resources of these marshes became a very urgent matter and/or recourse to reduce the areas targeted by inundation and being satisfied with the areas that can be sustainable and maintain the current status of the rest of the regions as an emerging ecosystem characterized by lands that are inundated every few years. Accordingly, steps must be urged to develop plans and programs to maintain the sustainability of these emerging ecosystems within the frameworks of climate change and the conditions of scarcity of water resources and water and air pollution to ensure that they are not lost in the future.

## Introduction

Marshlands are integrated ecological systems that contribute effectively to maintaining balance in the global environment system in addition to the economic and social importance of these areas. Wetlands comprise 5–8% of the earth’s land surface and about 20–30% of the earth’s carbon pool (Mitsch et al., 2013). Approximately 35% of these wetlands were lost up to 2015 with steadily increasing from 2000 (UN-Water, 2021).

Maintaining the continuity of life in these areas requires providing the water demands in quantity and quality that ensure the continuity of the balance of the ecosystems of these areas. Any damage to these areas will directly affect the general environmental, economic, and social system, which already suffers from many problems.

**Fig. 1:**
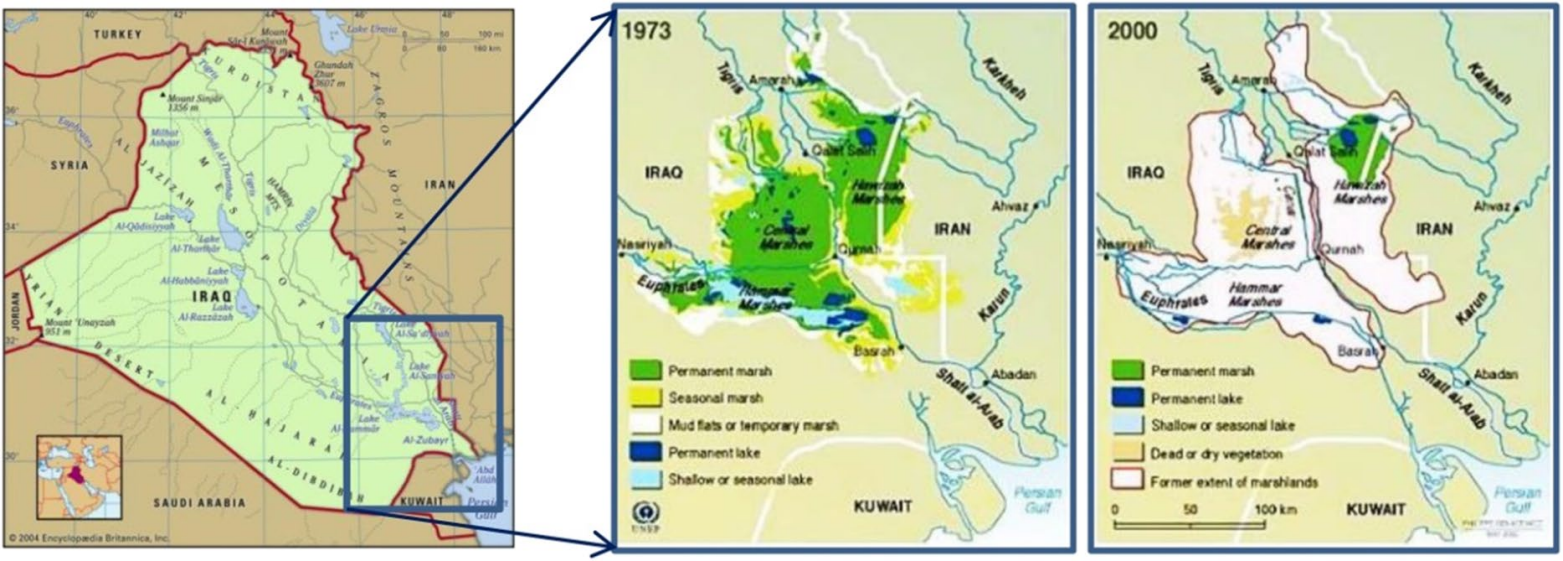
Location of the main Iraqi marshes

The Iraqi marshlands comprise the largest wetland ecosystem in the Middle East and Western Eurasia (Al-Handal & Chuanmin, 2015). They form an imperative part of the flyways and temporary habitat for migratory birds, support endangered species, and sustain freshwater fisheries, as well as the marine ecosystem of the Arab Gulf. It played a vital role in the social and economic advancement of the Iraqi people. The main Iraqi marshes are the Central Marshes (3121 km^2^), Hammar Marsh (2729 km^2^), and Hawizeh Marsh (3717 km^2^). These marshes are located in Missan, Thi-Qar, and Basrah Governorates. This marshland extended between latitude 29° 58′ 12″ N to 32° 30′ 0″ N and longitude from 45° 47′ 60″ E to 47° 42′ 0″ E. They accounting a total area of approximately 10,000 km^2^, as recorded in 1973 (Fig. 1) (Iraqi Ministries of Environment et al. 2006 unpublished study). Hawizeh Marsh is divided into two portions; the largest portion (79%) lies in Iraq while the rest (21%) known as Hor al-Azim Marsh is in Iran. These marshlands were the main contributor to the food and economic income of approximately half a million people in Iraq (Bedair et al., 2006). The draining of the marshes, which started during the Iraq-Iran war in 1980, has adversely impacted the ecosystem and displaced thousands of people. By the year 2000, only approximately 10% of these marshlands remain active (Al-Saboonchi et al., 2012). Al-Nussairi and Khalida (2020) pointed out that the total area of the marshes was reduced to less than 2000 km^2^ in 2003. The Central and Hammar Marshes vanished by 97% and turned to land covered with a salt crust, whereas only 34% of the Hawizeh Marsh area remained for military purposes (The Marine Science Centre of the University of Basra cited in Al-Nussairi and Khalida (2020).

Since 2003, concerted efforts have been put by mainly the Iraqi Ministry of Water Resources (IMoWR), the Ministry of Environment and the Ministry of Agriculture, civil society associations, non-governmental organizations, and the international community to assess the socio-economic and environmental conditions of the marshlands and the restoration requirements (Iraqi Ministries of Environment et al. 2006).

Literature shows that considerable research work was conducted on marsh restoration (Curtis et al., 2005; DAI, 2006; Iraqi Ministries of Environment et al. 2006; CRIM 2007 unpublished report; CRIM 2008 unpublished report; Riyadh et al., 2008; CRIM 2009a unpublished report; CRIM 2009b unpublished report; Al-Saboonchi et al., 2011; Al-Ameri and Jassim (2011); Al-Ansari & Knutsson, 2011; Al-Ansari et al., 2012; Muhannad et al., 2013; Al-Gburi et al., 2017; Al-Khafaji et al., 2018; Al-Khafaji et al., 2021; Bayda et al., 2022). Furthermore, various studies have shown that Iraq is one of the most vulnerable countries to climate change and its water resources begin significantly impacted, such as Chenoweth et al. (2011), Abbas et al. (2016), Abbas et al. (2017), Awchi and Kalyana (2017), Al-Khafaji and Al-Chalabi (2019), Constantinidou et al. (2019), and Saeed et al. (2021).

However, these studies were based on data sets with missing records that largely impaired the conclusions and recommendations. In addition, the marsh restoration in a changing climate was not carefully treated and well considered. Most of these studies were very ambitious and based on the optimistic hypothesis that the water needed to restore up to 75% of the marshes’ historical boundaries can be guaranteed. One key challenge facing the marshes is sustaining the freshwater input to maintain a healthy marshland system.

The extent of the inundated areas is subject to seasonal variation during the year and the type of hydrological year. Dry years lead to a decrease in the extent of marshlands, while wet years contribute to an increase in the inundated areas. Iraq is fed by the Tigris and the Euphrates Rivers; both originate outside of Iraq. These two rivers account for 98% of Iraq’s surface water supply (FAO 2017). Their flow is therefore very vulnerable to dams and water diversion networks in Turkey, Syria, and Iran. The key question is that “will water needed for health restoration and sustainable management of marshlands be available in the future, given the existing and planned dams, water diversions, and massive exploitation of water especially in Turkey and Iran, as well as competing water uses within Iraq?” The Güneydoğu Anadolu Projesi (GAP) in Turkey, a massive dam-building scheme of 22 dams and 19 hydropower plants across the Tigris-Euphrates basin with hydraulic diversion system and extensive irrigation development scheme, has significantly reduced the flow of water in downstream areas of both the Tigris and Euphrates Rivers (Al-Ansari, 2013; Al-Ansari & Sven, 2011; Altinbilek, 2004; Issa et al., 2014; Katalyn et al., 2013). In the last decade, the Center for Restoration of Iraqi Marshes and Wetlands (CRIMW) conducted site visits to Hawizeh Marsh, Central Marshes, and Hammar Marsh. On many occasions, this survey was hindered for security and financial reasons.

Various techniques such as remote sensing, Geographical Information System (GIS), and satellite imagery–based investigation by using spectral indices have been commonly used to assess the spatiotemporal variability of land cover for different types of ecological systems (Tian et al., 2019; Guo et al., 2017). The indices associated with these studies are the Normalized Difference Vegetation Index (NDVI), the Enhanced Vegetation Index (EVI), the Soil-Adjusted Vegetation Index (SAVI), the Modified Soil-Adjusted Vegetation Index (MSAVI), the Normalized Difference Moisture Index (NDMI), and the Water Index (WI) (for more details about these indices, see Jeffrey et al. (2006) and Eric et al. (2016)). Such indices were extensively utilized for spatiotemporal monitoring and assessment of the Iraqi water resources and marshlands (Abdul Razzak et al., 2010; Abdulmalik et al., 2021; Bayda et al., 2021; Becker, 2014; Francesca et al., 2009; Israa et al., 2011; Jones et al., 2008; Khamis et al., 2022; Mathew et al., 2016; Orkan et al., 2017; Shahad et al., 2022; UNEP, 2006).

This paper aims to investigate the effects of potential future water availability on the reality of applying the sustainable management plans of the Iraqi marshlands, subsequently specifying the deficit in preserving the water demand and the deviations from the planning levels of restoration. To this end, the recorded data of the available water resources, the inflow of water into the marshes, and the associated inundation marshland areas, as well as satellite images and the expected future decrease in the available water resources, were used to evaluate the present and expected situations of the marshland restoration. Moreover, the deviation of the present and future restoration from the planned levels was specified. Accordingly, the realism of applying the restoration plan of the Iraqi marshland was specified. The finding of this paper contributes to highlighting the most important challenges facing the sustainability of marshlands in arid and semi-arid regions, especially in the Middle East, under the effects of water scarcity due to climate change. This is very important for determining the right planning frameworks, making strategic decisions toward the sustainability of wetlands, and framing international cooperation on this issue.

## Method and materials

The balance between available water resources and demand within the economic and environmental constraints is a key to sustainable management of water resources, particularly in arid and semi-arid regions where water is limited in a changing climate. Balancing the development of Iraqi marshes requires (i) understanding the impact of climate change on water availability and water quality, (ii) estimating the magnitude and spatiotemporal distribution of water use needed to meet marsh restoration and sustainable development, (iii) understanding the current imbalance and the predicted gap between available water resources and increased demand, and (iv) understanding the environmental, economic, and social issues associated with the development and restoration of marshes areas.

This article aimed to carry out a water balance comparative study between the current and future marshes’ restoration and development and propose some recommendations to support the sustainable development of the marshes. To this end, the records of the available water resources and the supplied water to marshlands during the period 2009–2020 were analyzed and compared with the restoration requirement to examine the fulfillment level of restoration plan and specify the maximum, average, and minimum re-inundation areas (Re) of the marshes. Satellite imagery–based investigation was conducted by using NDVI, NDMI, and WI to compute the vegetation cover and Re for each marsh to assess the viability of the inundation process and the effectiveness of restoration plans.

Moreover, data on water demand for all water consumption sectors including irrigation, domestic, industrial, environmental, and marshlands for the same period was gathered and assessed according to the supplied water to marshlands and available water resources. The potential situation of water resources in Iraq such as the expected decrease in water resources and increase in water demand, due to climate change, population growth, and heavy exploitation of upstream countries (Turkey, Iran, and Syria) to water resources, was also examined to evaluate the expected deficit in preserving the restoration requirement and the constituent challenges. Based on this deficit and available water and according to the relationship between the available annual water volume and percentage of inundation area to total marsh area for the three marshes, the expected decrease in the restorable areas of Iraq marshes was calculated. Consequently, the expected situation of the restoration level corresponding to the potential deficit in water resources was estimated. Accordingly, the realism of applying the sustainable management plans of the Iraqi marshlands was assessed and discussed to obtain a definitive conclusion regarding the possibility of implementing restoration plans for the Iraqi marshlands and reliance on achieving the sustainable management of these ecosystems.

### Study area

The marshes in Iraq (Fig. 1) are naturally subjected to extensive seasonal and annual variations depending on the hydrological conditions (flood, dry, or normal year). Between 2003 and 2006, the Iraqi Ministries of Environment, Water Resources and Municipalities and Public Works in cooperation with the Italian Ministry for the Environment and Territory, Free Iraq Foundation, and the UN Environment Programme (UNEP) discussed four scenarios of recuperation Iraqi marshes (0–25%, 25–50%, 50–75%, and 75–100%) of the former marshlands’ coverage (Iraqi Ministries of Environment et al. 2006).

The Hor El Azim Marsh, which is the Iranian part of the Hawizeh Marsh, has been hydrologically separated from the Iraqi part of the marsh, especially during the moderate and dry water years. Also, it is currently not listed in the cultural heritage sites by UNESCO (visit UNESCO World Heritage Centre-World Heritage List) and not included in the Ramsar Convention on Wetlands (visit Country profiles | Ramsar). Therefore, this part of the marsh was not considered in this paper.

**Fig. 2:**
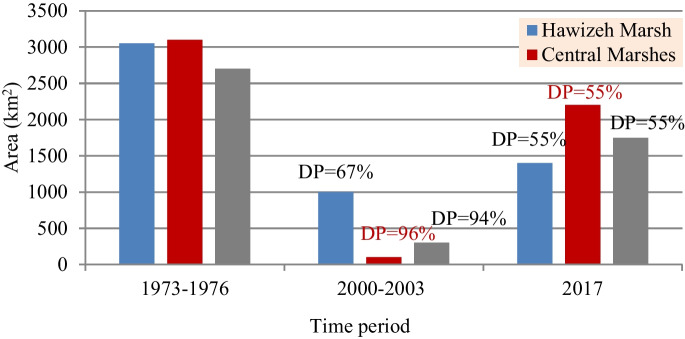
Areas of main Iraq’s marshlands for the periods 1973–1976, 2000–2003, and 2017

Figure 2 shows the area of the three marshes as observed in the reference period (1973–1976), the shrinkage rate recorded between 2000 and 2003 due to drying processes, and the recuperation rate in 2017. The three marshlands were designated as wetlands of international importance and protected under the Ramsar Convention in February 2008 (visit Country profiles | Ramsar) and recently added to UNESCO’s World Heritage List (visit UNESCO World Heritage Centre-World Heritage List). According to the Strategic Study for Water and Land Resources in Iraq (SWLRI) (IMoWR 2014 unpublished study), the total restorable area of the marshlands accounts for 60% (5600 km^2^) of the total area recorded in 1973 (10,000 km^2^) except for Iranian part of the Hawizeh Marsh (21% of 3717 km^2^). This area is divided into 1377 km^2^, 1762 km^2^, and 2420 km^2^ for the Hawizeh, Hammar, and Central Marshes, respectively.

### Water feeding sources of the marshlands

The main sources of water feeding the marshes are those that come from the Tigris River, Euphrates River, and Shatt Al-Arab River as well as Karkah, al-Teeb, and Duwereg rivers from Iran. The Hawizeh Marsh is fed by two branches of the Tigris River (al-Musharrah and al-Kahlaa) in Iraq. The marsh also receives flood water during flood years through Kumait flood escape in Iraq. Three rivers (al-Karkheh, al-Teeb, and Dwairege), which originate in Iran, are discharging into the marsh. The Hammar Marsh takes the water through a system of Euphrates River branches in Iraq (Um-Naklah, Haffar, Bani Hassan, Eglaiwen, Ekaika, Safha, and Karmat Bani Seed). In flood season (during flood years), this marsh receives water from the Central Marshes through several orifices and spillways on the existing dykes between these marshes. Tigris River is the only feeder of the Central Marshes. Butaireh and al-Majar El Kabeer Rivers are the principal feeders of this marsh from the Tigris River (Iraqi Ministries of Environment et al. 2006).

### Available water resources

**Fig. 3:**
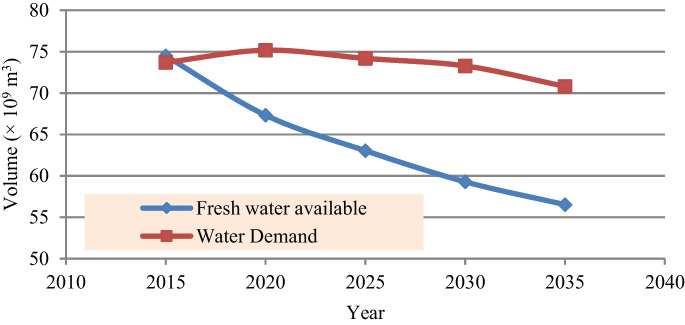
Comparison of fresh water available and water demand in Iraq for the period 2015 to 2035 (IMoWR 2014)

Water resources in Iraq are highly fluctuated from year to year. Data recorded between 1995 and 2020 shows that the average total annual available water resources in Iraq were approximately between 81 × 10^9^ and 85 × 10^9^ m^3^ (IMoWR (2014); Chabuk et al., 2020; Al-Ansari (2021). While surface water accounts for 85 to 89% of these resources, the remainder is drainage and groundwater. Most of the surface water, approximately 80 × 10^9^ m^3^, comes from the Tigris and Euphrates Rivers. Most water of these rivers, 77%, comes from Turkey. While 23% comes from Iran, Syria, and Iraq with rates of 13, 8, and 2%, respectively (IMoWR 2014; Al-Ansari, 2021). However, climate change, the increase of domestic and industrial water demand, construction of numerous dams, and irrigation project by neighboring countries is expected to decrease the available water resources by 2035 to approximately 25% (Altinbilek, 2004; Katalyn et al., 2013; IMoWR 2014). Figure 3 illustrates the projected fresh water available and water demands by 2035 (IMoWR 2014).

#### Tigris River

River Tigris is the second-largest river in western Asia. It rises in the southeastern part of Turkey. Its watershed includes four countries Turkey, Iran, Syria, and Iraq. The mean discharge of the river at Mosul City before 1984 was 701 m^3^/s (Mohamed et al., 2016). Most previous studies, such as World Bank (2006), Issa et al. (2014), and Al-Ansari et al. (2019), indicated that the mean annual inflow of the Tigris into Iraq is ranging from 20 × 10^9^ m^3^ to 23 × 10^9^ m^3^. Within Iraq from Fishkhabour at the north to south of Baghdad, additional inflow is received from the river tributaries (five tributaries) that reach 25 to 29 × 10^9^ m^3^ (Al-Ansari & Knutsson, 2011). For the period 1932 to 2008, the annual average of the river was 49.48 × 10^9^ m^3^, of which 56% comes from Turkey. The remainder 44% of which 12% comes from Iran and 32% originates in Iraq (IMoWR 2014; Al-Ansari, 2021). Aliso Dam, which is the largest dam on the Tigris River in Turkey with a storage capacity of 10.4 × 10^9^ m^3^, which operated in 2019, is expected to decrease the annual average inflow of the Tigris River to 39.84 × 10^9^ m^3^ (Al-Madhhachi et al., 2020; IMoWR 2014; Issa et al., 2014).

#### Euphrates River

The long-term mean annual flow average inflow of the Euphrates River into Iraq decreased from 35 × 10^9^ to 24 × 10^9^ m^3^ during the period from 1970 to 2003. Most of the Euphrates water comes from Turkey (88%) while Syrian and Iraq contributions are 9% and 3%, respectively (IMoWR 2014; Issa et al., 2014). In many years, the inflow decreased to 10 × 10^9^ m^3^/year, which is approximately 30% of the long-term mean annual flow. This significant decrease in flow volume can be mainly attributed to the Southeastern Anatolia Project (Turkish: Güneydoğu Anadolu Projesi, GAP) that is started in 1970 and has continued till now. Moreover, Syria has serious plans for development and hopes to double the irrigated land from the Euphrates to reach 7400 km^2^, which increases the net water used from 5 × 10^9^ to 10 × 10^9^ m^3^/year. By completing the development process, Turkish and Syrian projects could reduce Iraq’s share of the Euphrates from 24 × 10^9^ m^3^/year to only 9 × 10^9^ m^3^/year. In drought years, Iraq’s share will fall from 75 to 28% (IMoWR 2014; Issa et al., 2014). Accordingly, the present and expected future inflow of the Euphrates River into Iraq is 28.27 × 10^9^ m^3^/year and 11.84 × 10^9^ m^3^/year, respectively.

#### Shatt Al-Arab River

The Shatt Al-Arab River extends from the north to the south of the Basrah Governorate where its outfall is in the Arabian Gulf. The water source of this river comes from the Tigris and Euphrates Rivers within Iraq and the rivers of Karkheh (through Hawizeh Marsh and then Swaib River) and Karon flow within Iranian lands. The average annual flow of the Shatt Al-Arab River at its outfall to the Arabian Gulf in the Fao City was 37.5 × 10^9^ m^3^ (1189 m^3^/s) in the years 1977and 1978. In 1995 and 2008, the annual flow decreased to 25 × 10^9^ m^3^ (815 m^3^/s) and 8 × 10^9^ m^3^ (246 m^3^/s) respectively. In 2010, the flow of the Shatt Al-Arab River was completely dependent on the flow from the Tigris River only with a mean annual flow of 1.8 × 10^9^ m^3^ (58 m^3^/s), with a monthly variation limited from 42 to 90 m^3^/s. during October and May respectively (Al-Asadi, 2017; Al-Asadi & Abdulzahra, 2019). The water flow in the Shatt Al-Arab River is very complex because it is affected by the tidal phenomenon of the Arabian Gulf where the river experiences a tidal cycle of approximately 13 h (Abdullah, 2017). For the years 2019 and 2020, the annual average inflow during the tide (ebb) periods from (to) Shatt Al-Arab River to (from) the Hammar marsh through Karmat Ali River was about 230 m^3^/s (200 m^3^/s) (CRIMW, 2021 unpublished data).

### Water use and demands

In Iraq, the water is withdrawn for municipal, industrial, agricultural, fish farms and livestock, and marshlands use. The average annual total water demand for the period from 1970 to 1990 was approximately 43 × 10^9^ m^3^/year (Neda, 2008). This demand gradually increased to around 52 × 10^9^ m^3^/year in 2003. However, most of the water demands, 90%, are consumed in the agricultural sector (World Bank, 2006). After 2003, IMoWR allocated 11× 1 0^9^ m^3^/year for restoring the marshlands. Climate change and population growth with uncontrolled, unattended, and inefficient use increased the total water demand to approximately 74 × 10^9^ m^3^/year in 2015. However, the annual average total water demand is expected to range between 70 × 10^9^ to 75 × 10^9^ m^3^/year during the period from 2020 to 2035 (IMoWR 2014).

Currently, the annual average water demand of the marshlands is 9.25 × 10^9^ m^3^/year. This amount is required to maintain the current area (3600 km^2^) of these marshes, which accounts for 64% of the total restorable area of the marshlands (5600 km^2^) (IMoWR 2014). This demand account for 12% and 18% of the present and future total annual average inflow of the Tigris and Euphrates Rivers, which is 77.75 × 10^9^ and 51.32 × 10^9^ m^3^/year, respectively. Iraqi marshland restoration plan aims at restoring 75%, which is 4200 km^2^, of the total restorable area and sustaining the ecological systems of these marshlands according to international standards. The required annual average water demand to achieve this aim is 11 × 10^9^ m^3^/year (Iraqi Ministries of Environment et al. 2006). This amount constitutes 14% and 21% of the present and future total annual average inflow of the Tigris and Euphrates Rivers.

#### Water demand of Hawizeh Marsh

**Fig. 4:**
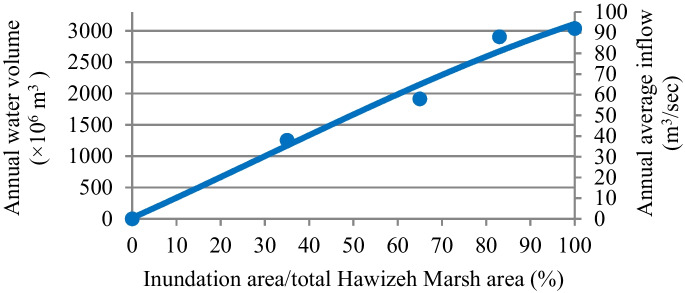
Water demand for the restoration of Hawizeh Marsh (Iraqi Ministries of Environment et al. 2006)

The annual average water demand of Hawizeh Marsh for restoring 65 to 75% (1908 km^2^ to 2200 km^2^) of the total area of this marsh is 2.0 × 10^9^ to 2.4 × 10^9^ m^3^/year (see Fig. 4) (Iraqi Ministries of Environment et al. 2006). Most of this demand was supplied from Iran through the Karkah, al-Teeb, and Duwereg rivers. For restoring 65 to 75% of the marsh, the annual average water demands of this marsh from Tigris River are 0.78 × 10^9^ to 1.03 × 10^9^ m^3^/year. These amounts constitute 1.58 to 2.08% of the present annual average inflow of the Tigris River. While they constitute 1.96 to 2.58% of the future inflow (Fig. 4).

#### Water demand of Hammar Marsh

**Fig. 5:**
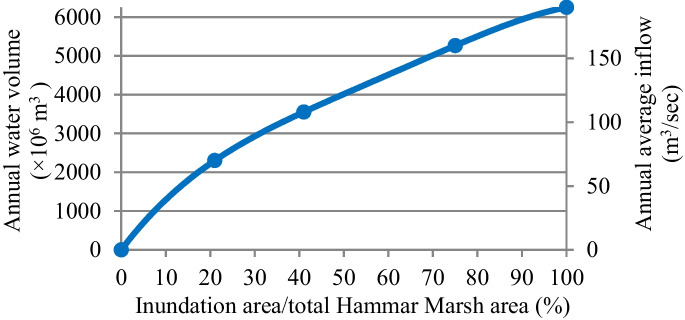
Water demand for the restoration of Hammar Marsh (Iraqi Ministries of Environment et al. 2006)

Restoring 55% (1501 km^2^) to 75% (2047 km^2^) of the total area of the Hammar Marsh requires annual average water discharge of 4.0 × 10^9^ to 5.0 × 10^9^ m^3^/year, respectively (see Fig. 5) (Iraqi Ministries of Environment et al. 2006). All these demands were supplied by the Euphrates River. These discharges account for 14.15 to 17.69% of the present annual average inflow of the Euphrates River. While they constitute 33.78 to 42.23% of the future inflow (Fig. 5).

#### Water demand of Central Marshes

**Fig. 6:**
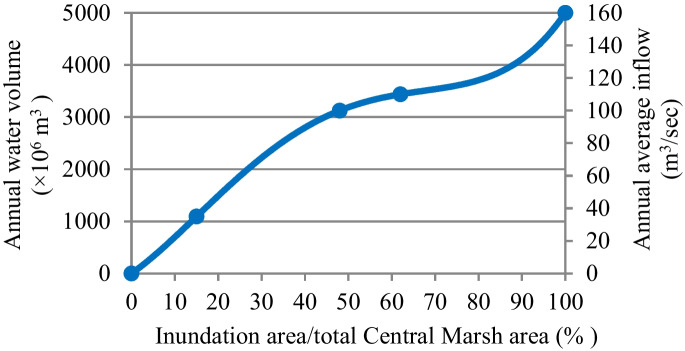
Water demand for the restoration of the Central Marshes (Iraqi Ministries of Environment et al. 2006)

Tigris River is the only water source of the Central Marshes. The average annual required discharge from this river to restore 50% (1561 km^2^) to 75% (2341 km^2^) of the total area of this marsh is 3.25 × 10^9^ to 3.6 × 010^9^ m^3^/year, respectively (Iraqi Ministries of Environment et al. 2006). These discharges constitute 6.57 to 7.27% of the present annual average inflow of the Tigris River. While they constitute 8.16 to 9.04% of the future inflow (see Fig. 6).

### Present situation of marsh restoration

For the past ten years (2009–2020), the eligible areas for restoration according to the CRIMW plan specified by (Iraqi Ministries of Environment et al. 2006) are 1377 km^2^, 1762 km^2^, and 2420 km^2^ for the Hawizeh, Hammar, and the Central Marshes, respectively. These areas were targeted to restore and from which the restoration proportions are extracted. During these years, CRIMW conducted field works (in 28 locations within the marshes areas) to monitor the inundation and restoration level of the marsh area and ecological system including investigation and assessment of the inundated area, land cover, water quality, and the main biological and ecological aspects of the marshes. The monthly re-inundated (Re) areas and the inflow into the marshes over the period 2009 to 2020 are given in Tables 1, 2, and 3 (CRIMW, 2021, unpublished data).

**Table 1:** Re-inundated area (Re) of and inflow into Hawizeh Marsh over the period 2009–2020 (CRIMW, 2021 unpublished data)

Year	Month											
	Jan	Feb	Mar	Apr	May	Jun	Jul	Aug	Sep	Oct	Nov	Dec
Area (km^2^)												
2009	941	1021	997	951	896	820	718	594	635	616	620	619
2010	592	642	659	660	663	717	676	703	682	649	643	672
2011	733	734	763	780	764	770	793	750	712	687	672	649
2012	611	214	634	665	648	599	584	542	532	533	535	590
2013	611	214	634	665	648	599	584	542	532	533	535	590
2014	736	870	1025	1079	959	859	1121	1055	932	749	895	799
2015	847	717	615	831	917	831	651	507	395	433	553	399
2016	507	476	619	617	685	732	641	619	601	621	465	424
2017	473	455	538	646	822	749	674	470	413	398	335	338
2018	371	417	448	478	466	420	410	381	290	319	574	965
2019	372	361	426	455	422	469	433	466	512	390	422	368
2020	383	372	365	347	401	455	448	455	451	401	415	361
Inflow (m^3^/s)												
2009	21.2	31.4	28.4	33.4	46.9	18.2	14.2	9.8	11.2	8.4	12.0	20.5
2010	0.0	0.0	10.5	21.2	24.4	15.3	13.3	12.2	11.5	10.9	9.2	7.0
2011	7.6	17.5	6.8	6.4	14.0	11.8	11.0	8.0	15.5	7.2	12.3	12.0
2012	10.9	14.5	15.5	12.0	19.0	15.5	20.4	16.0	12.3	11.4	7.0	26.2
2013	55.0	150.4	77.0	53.3	160.5	47.5	26.5	19.5	29.0	14.8	48.5	16.5
2014	43.5	51.5	50.0	93.5	73.0	25.0	24.0	17.0	16.5	13.5	4.5	0.0
2015	5.2	8.4	6.7	17.0	22.3	9.5	2.0	0.0	3.0	5.0	25.3	16.9
2016	42.2	26.3	57.8	59.0	42.3	15.5	21.3	10.5	10.0	10.5	0.5	8.3
2017	24.2	8.8	23.7	28.3	58.3	19.3	8.4	2.5	1.5	1.5	0.0	0.1
2018	2.2	5.0	7.5	9.9	7.9	4.3	1.3	2.3	1.8	1.1	0.5	97.9
2019	20.4	17.5	35.1	43.1	34.3	46.9	36.8	45.6	59.5	24.6	34.3	18.7
2020	23.4	20.0	17.9	12.9	27.6	43.1	40.6	42.7	42.3	27.6	31.8	17.5

**Table 2:** Re-inundated area (Re) and inflow into Hammar Marsh over the period 2009–2020 (CRIMW, 2021 unpublished data)

Year	Month											
	Jan	Feb	Mar	Apr	May	Jun	Jul	Aug	Sep	Oct	Nov	Dec
Area (km^2^)												
2009	850	1032	990	877	887	639	517	300	349	314	379	410
2010	529	618	729	676	723	750	753	886	945	969	1070	1156
2011	1224	1302	1314	1264	1280	1206	1328	1243	1185	1111	1176	1228
2012	1344	1443	1463	1306	1289	1186	1173	1214	1190	1225	1232	1391
2013	1344	1443	1463	1306	1289	1186	1173	1214	1190	1225	1232	1391
2014	1610	1429	1363	1048	1380	1342	1162	1300	1397	1543	1286	1525
2015	1361	1397	1450	1291	1387	1274	916	897	772	774	813	791
2016	1110	1202	1262	1259	1270	1307	1296	1186	1302	1155	1111	1164
2017	1197	1133	1251	1293	1381	1174	1150	929	809	772	619	620
2018	713	829	869	890	868	784	739	608	514	500	638	956
2019	638	589	884	1018	870	1081	912	1060	1292	708	870	610
2020	687	631	596	511	757	1018	976	1011	1004	757	828	589
Inflow (m^3^/s)												
2009	55.2	53.4	41.7	58.3	50.5	63.5	43.8	46.6	49.9	66.5	42.4	58.6
2010	0.0	3.7	3.5	4.4	8.3	4.6	6.3	5.7	19.4	46.9	31.8	24.7
2011	53.3	92.4	58.1	86.0	69.0	54.5	74.0	81.3	68.0	66.5	36.9	42.4
2012	56.2	61.9	45.5	57.8	56.2	62.0	74.6	80.1	86.1	83.1	52.5	67.2
2013	121.3	100.9	54.6	82.7	69.0	56.7	59.5	67.6	82.6	96.5	96.4	70.4
2014	60.0	57.0	32.7	50.1	54.3	45.0	49.4	72.3	58.6	68.6	39.7	47.4
2015	43.3	46.3	33.4	64.3	44.7	22.8	7.4	13.3	25.4	27.3	24.2	21.0
2016	65.5	45.4	54.9	70.9	58.1	47.9	56.6	42.6	59.2	56.9	22.9	41.0
2017	55.6	32.4	41.2	61.1	54.3	59.0	53.2	43.9	37.8	33.1	15.2	16.6
2018	41.3	41.1	51.3	47.2	40.8	19.2	12.9	12.2	12.3	19.2	62.3	96.3
2019	48.2	41.5	83.3	52.0	61.7	46.2	78.3	81.6	41.1	58.2	81.4	44.1
2020	54.8	47.4	42.2	30.3	65.6	20.1	96.2	101.4	97.6	65.0	75.0	41.5

**Table 3:** Re-inundated area (Re) of and inflow into the Central Marshes over the period 2010–2020 (CRIMW, 2021 unpublished data)

Year	Month											
	Jan	Feb	Mar	Apr	May	Jun	Jul	Aug	Sep	Oct	Nov	Dec
Area (km^2^)												
2009	354	415	416	381	315	184	185	89	117	94	122	122
2010	320	373	341	345	322	370	345	449	389	407	407	466
2011	559	579	563	480	488	511	506	419	420	445	446	514
2012	505	674	748	562	548	564	526	490	480	505	507	703
2013	505	674	748	562	548	564	526	490	480	505	507	703
2014	1338	1489	1621	1442	1262	1358	1241	1035	1023	978	1010	1077
2015	1152	1340	1220	1244	1257	1098	764	541	410	431	419	375
2016	719	748	786	872	1153	1162	1123	1009	1095	841	804	925
2017	1143	1000	1198	1344	1478	1247	1188	1103	982	925	808	810
2018	942	1047	1108	1146	1121	974	939	810	707	683	852	1270
2019	661	635	789	860	782	893	804	881	1003	698	782	646
2020	687	658	639	595	723	860	837	856	852	723	760	635
Inflow (m^3^/s)												
2009	46.3	48.0	47.2	51.2	67.5	62.9	39.6	34.0	70.9	45.2	35.3	44.2
2010	2.9	10.4	8.4	18.4	22.5	11.1	15.8	11.2	34.9	35.5	24.9	24.7
2011	26.9	46.8	20.3	22.1	40.5	40.1	40.6	36.2	51.5	56.0	38.2	34.5
2012	35.7	44.2	35.3	37.9	62.8	39.5	47.1	47.0	43.3	45.0	19.6	50.9
2013	78.6	92.6	82.5	49.9	101.5	48.8	51.9	48.1	66.1	63.6	79.6	58.0
2014	110.7	60.7	89.1	96.5	92.1	59.8	61.8	52.7	45.5	35.8	19.1	26.7
2015	39.4	52.1	26.3	42.2	53.7	19.4	17.3	11.4	16.5	13.5	35.0	38.3
2016	51.1	58.6	81.0	73.0	94.5	72.0	57.5	46.8	53.6	48.6	21.2	41.5
2017	48.5	35.8	43.1	69.4	98.6	71.8	48.0	33.8	31.3	22.2	8.5	9.3
2018	22.8	30.8	38.5	51.8	41.1	23.5	16.3	18.8	15.6	13.6	71.8	113.8
2019	44.6	39.9	68.4	81.4	67.1	87.5	71.2	85.4	107.9	51.4	67.1	41.9
2020	49.4	44.0	40.6	32.4	56.2	81.4	77.3	80.7	80.0	56.2	63.0	39.9

### Satellite imagery–based investigation for the present situation of marsh restoration

**Fig. 7:**
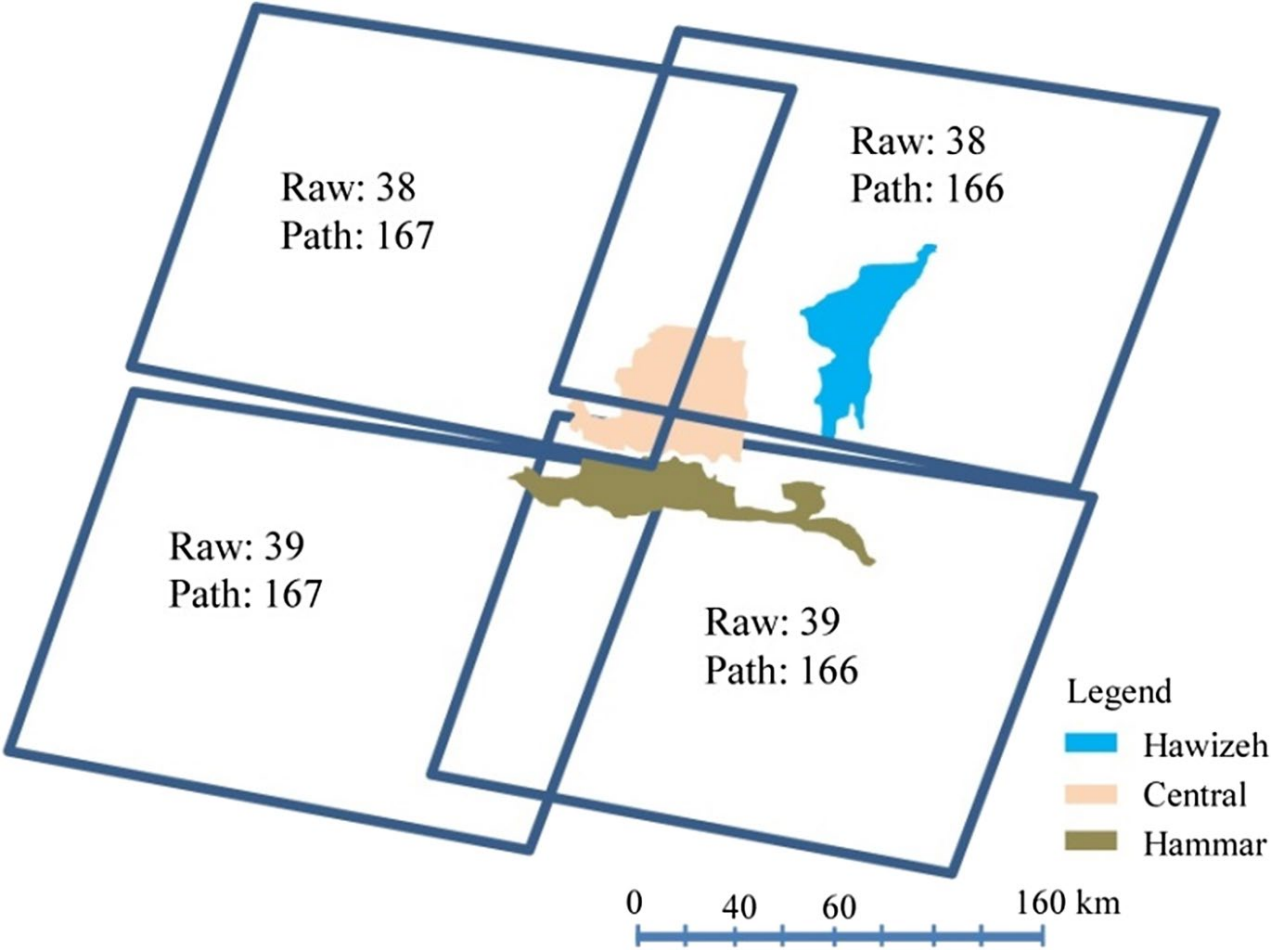
Location of the Hawizeh, Hammar, and the Central marshlands concerning the WRS-2 Landsat 7 and 8 swaths

Satellite imagery data is optimal to provide comprehensive coverage for the large areas of the Iraqi marshlands and overcoming the problem of the lack of continuous access to some marshland regions. Satellite images of the Landsat 7 (L7) and 8 (L8) sensors for the period 2009 to 2020 were used to evaluate the spatiotemporal change in the land cover of the marshlands as well as vegetation health. The surface reflectance and top of atmosphere (TOA) images, which use the USGS-WRS-2 system, were downloaded from the USGS website (http://earthexplorer.usgs.gov) (see Fig. 7). For all the downloaded images, the necessary preprocessing such as atmospheric, geometric, and terrain correction was implemented. Furthermore, the failure of the scan line corrector in the ETM+ sensor of the Landsat 7 images was corrected. Additionally, censor-affected sections were processed by using quality and cloud masks of cloud confidence, cloud shadow, and snow. Subsequently, the images for a given date were stacked and then clipped for each marsh. Image processing was performed by using the ArcGIS and Raster packages in the R software (Richard, 2018). For each marshland, a series of satellite imagery–based indices, which are NDVI, NDMI, and WI, was computed and used to assess the spatiotemporal change in the land cover and vegetation health of the marshlands. These indices are based on the spectral bands 2 to 6 (B2, B3, B4, B5, and B6) of the satellites L7 and L8. The spatial distribution of the index values was mapped utilizing the zonal statistics tool in ArcGIS software.

**Fig. 8:**
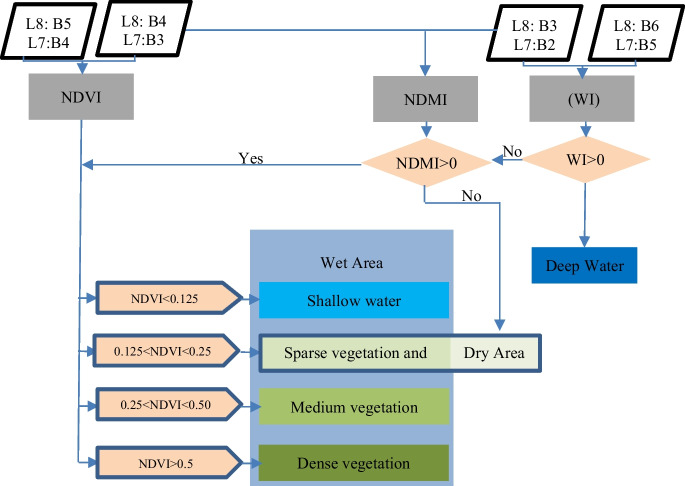
Methodology of identifying marshland extent, open waters, and vegetated areas

UNEP (2006) specified the NDVI thresholds for describing the vegetation status of the Iraqi marshlands. These thresholds stated that the land cover has dense vegetation, medium density vegetation, sparse vegetation, or vegetative cover if NDVI values are greater than 0.5, greater than 0.25, greater than 0.125, or less than 0.125, respectively. These thresholds were adopted in this research. However, Komeil et al. (2014) showed that wetted areas have NDMI greater than zero whereas Adrian et al. (2016), Lei et al. (2009), and Hanqiu (2006) showed that areas of WI greater than zero correspond to areas covered with water. Accordingly, the spatiotemporal distribution of land cover and vegetation statute within the marshlands was evaluated based on the methodology shown in Fig. 8.

## Results

### Fulfillment level of restoration plan

**Fig. 9:**
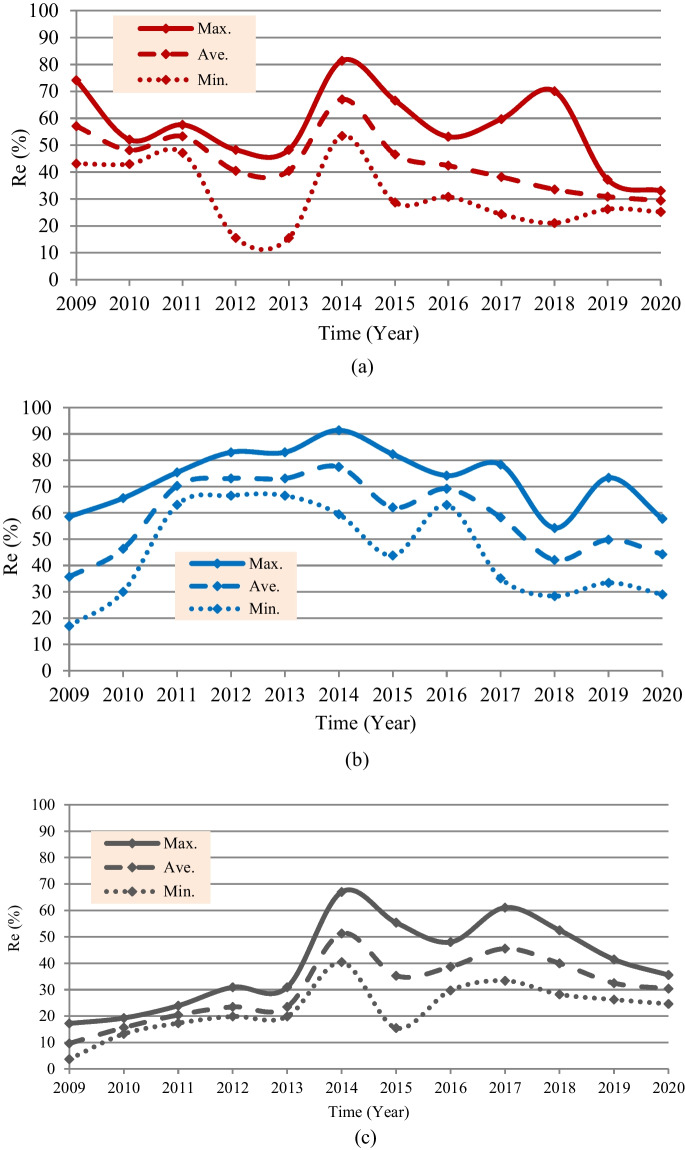
Re rates from the eligible restoration areas: **a** Hawizeh Marsh, **b** Hammar Marsh, and **c** Central Marshes

Based on the recorded data of the present situation of the marsh restoration for the period 2009 to 2020, as reported by CRIMW (Tables 1, 2, and 3), the maximum, average, and minimum Re of the marshes are listed in Table 4. Furthermore, the maximum, average, and minimum Re rates from the eligible restoration areas of Hawizeh Marsh (1377 km^2^), Hammar Marsh (1762 km^2^), and the Central Marshes (2420 km^2^) are as shown in Fig. 9. This figure shows that during the last 10 years, the maximum (minimum) Re rates of Hawizeh, Hammar, and the Central Marshes were 81% (16%), 91% (17%), and 67% (4%) from the eligible restoration areas. The maximum Re rate occurred in 2014. However, this high rate was maintained for just 1 year (2014); then, it decreased to less than 70%, 80%, and 60% for Hawizeh, Hammar, and the Central Marshes respectively. In general, the average Re rates of these marshes were 47%, 61%, and 30% respectively. It is worth mentioning that these rates represent 21%, 40%, and 23% of the original area (before the year 1980) of Hawizeh (3100 km^2^), Hammar (2700 km^2^), and Central Marshes (3200 km^2^) respectively.

**Table 4:** Maximum, average, and minimum Re-inundated area (Re (km^2^)) and inflow (*Q* (m^3^/s)) of the Marshes over the period 2009–2020

Year	Hawizeh Marsh						Hammar Marsh						Central Marshes					
	Max.		Average		Min.		Max.		Average		Min.		Max.		Average		Min.	
	Re	*Q*	Re	*Q*	Re	*Q*	Re	*Q*	Re	*Q*	Re	*Q*	Re	*Q*	Re	*Q*	Re	*Q*
2009	1021	46.9	786	21.3	594	8.4	1032	66.5	629	52.5	300	41.7	416	70.9	233	49.4	89	34.0
2010	717	24.4	663	11.3	592	0.0	1156	46.9	817	13.3	529	0.0	466	35.5	378	18.4	320	2.9
2011	793	17.5	734	10.8	649	6.4	1328	92.4	1238	65.2	1111	36.9	579	56.0	494	37.8	419	20.3
2012	665	26.2	557	15.1	214	7.0	1463	86.1	1288	65.3	1173	45.5	748	62.8	568	42.4	480	19.6
2013	665	160.5	557	58.2	214	14.8	1463	121.3	1288	79.9	1173	54.6	748	101.5	568	68.4	480	48.1
2014	1121	93.5	923	34.3	736	0.0	1610	72.3	1365	52.9	1048	32.7	1621	110.7	1240	62.5	978	19.1
2015	917	25.3	641	10.1	395	0.0	1450	64.3	1094	31.1	772	7.4	1340	53.7	854	30.4	375	11.4
2016	732	59	584	25.4	424	0.5	1307	70.9	1219	51.8	1110	22.9	1162	94.5	936	58.3	719	21.2
2017	822	58.3	526	14.7	335	0.0	1381	61.1	1027	42.0	619	15.2	1478	98.6	1102	43.4	808	8.5
2018	965	97.9	462	11.8	290	0.5	956	96.3	742	38.0	500	12.2	1270	113.8	967	38.2	683	13.6
2019	512	59.5	425	34.7	361	17.5	1292	83.3	878	59.8	589	41.1	1003	107.9	786	67.8	635	39.9
2020	455	43.1	405	29.0	347	12.9	1018	101.4	780	61.4	511	20.1	860	81.4	735	58.4	595	32.4

**Fig. 10:**
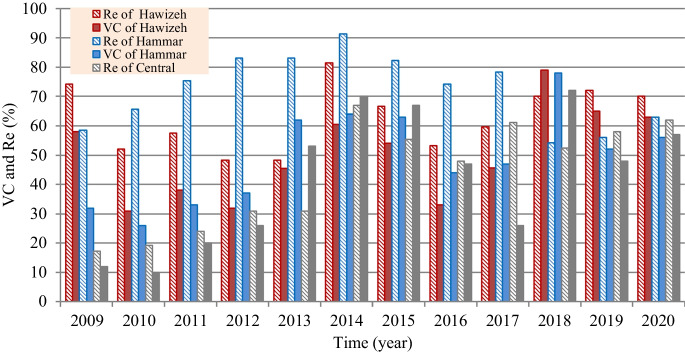
Vegetation cover and Re-inundated areas of the marshes for the period 2009 to 2020

**Fig. 11:**
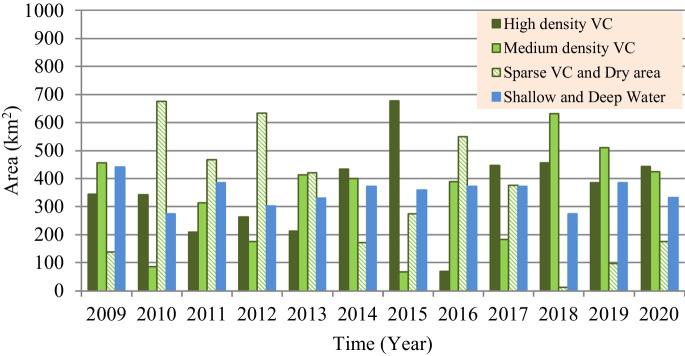
Land cover of Hawizeh Marsh for the period 2009 to 2020

**Fig. 12:**
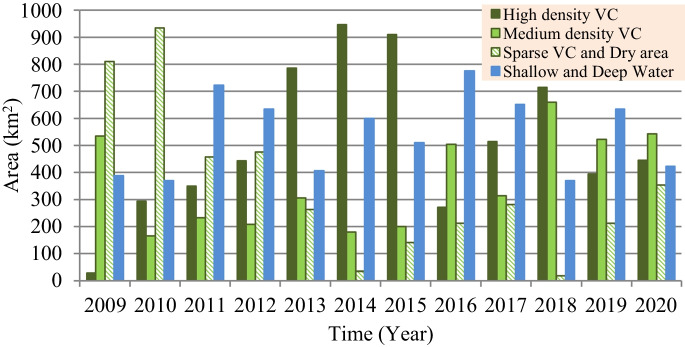
Land cover of Hammar Marsh for the period 2009 to 2020

**Fig. 13:**
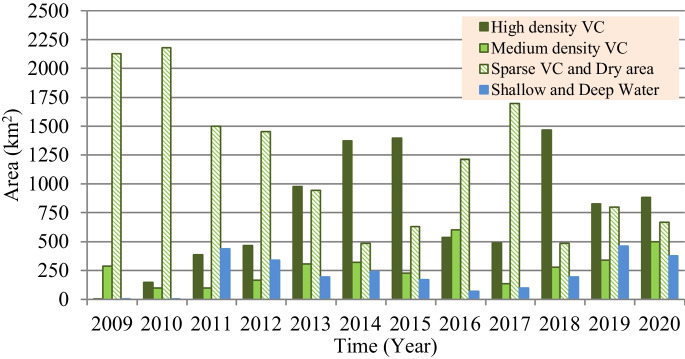
Land cover of the Central Marshes for the period 2009 to 2020

However, based on the satellite imagery–based investigation for the present situation of marsh restoration, percentages of the vegetation covers (VC) and Re are computed and presented in Fig. 10. Findings show that the maximum percentages of VC and (Re) were 79.0% (81.4%), 78.0% (91.4%), and 72.0% (67.0%) for Hawizeh, Hammar, and the Central Marshes, respectively. For the three marshes, the maximum Re was observed in 2014, whereas the maximum VC was in 2018. The increase in VC can be attributed to the relative stability of the inundation rates during the period 2014–2018 compared to the period before 2014. This indicates the importance of stabilizing the feeding of the marshes at rates within fluctuation limits that ensure the continued growth and preservation of VC. Also, the classification of the marshes’ land cover (Figs. 11, 12, and 13) indicated that the maximum (minimum) percentage of dense VC, medium VC, spread VC, and dry area and water area were 49% (5%), 46% (5%), 49% (1%), and 32% (20%) for Hawizeh Marsh; 54% (2%), 37% (9%), 53% (1%), and 44% (21%) for Hammar; and 60% (0.1%), 25% (4%), 90% (20%), and 18% (0.1%) for the Central Marshes. In Hawizeh Marsh, there was a slight increase in the dense and medium VCs, whereas enhancement in these classes was better in Hammar Marsh. In the Central Marshes, the situation fluctuated and there was no noticeable improvement in the areas of dense or medium VC, although the situation after 2014 was slightly better than the years before. However, despite the maximum Re of Hawizeh, Hammar, and the Central Marshes which was 81%, 91%, and 67%, respectively, which satisfy the targeted levels in the restoration plan shown in Figs. 6, 7, and 8, this high rate was maintained for just 1 year (2014). Accordingly, these insufficient and fluctuating inundation rates did not lead to achieving the objectives of restoration plans.

### Expected decrease in marshland water resources

As the annual average flow of Tigris and Euphrates is expected to decrease further by 2025 to more than 25% and 50%, respectively (Al-Ansari & Knutsson, 2011; Altinbilek, 2004; Katalyn et al., 2013), therefore, the average total annual flow of these rives is expected to be 51.25 × 10^9^ m^3^, with 37.11 × 10^9^ m^3^ (72.4%) from the Tigris River and 14.14 × 10^9^ m^3^ (27.6%) from the Euphrates River, the difference between these values and those shown in Fig. 3 is attributed to other water resources such as the groundwater. However, the annual average total water demand of various sectors (agriculture, municipal and industrial, marshlands, fish farm, and livestock and flow to the Arabian Gulf via Shatt Al Arab River) for the years 2025 and 2035 is expected to be 74.19 × 10^9^ m^3^ and 70.80 × 10^9^ m^3^ (IMoWR 2014) with a deficit of 11.15 × 10^9^ m^3^ (15%) and 14.28 × 10^9^ m^3^ (20%), respectively (Fig. 2). According to the share of each river from the total inflow, the deficit for the years 2025 and 2035 will be 8.07 × 10^9^ m^3^ (10.9% of the total) and 10.33 × 10^9^ m^3^ (14.60% of the total) for the Tigris River and 3.08 × 10^9^ m^3^ (4.15% from the total) and 3.94 × 10^9^ m^3^ (5.56% of the total) for Euphrates River.

The domestic and agricultural demands take the higher priority because of their direct impact on the life of most Iraqi people, because of their link to food security. Therefore, they should be insured, especially the domestic demand. Thence, the largest proportion of the deficit will involve the marsh sector. However, the hypothesis of distributing the deficit equally over all sectors (this can be named Scenario 1 (S1) and distributing the deficit according to the percentage of demand for each sector (Scenario 2 (S2)) leads to the application of the most optimistic scenarios that are less harmful to the marshes.

According to S1, the expected deficit and net available water for the considered marshes by the years 2025 and 2035 will be as listed in Table 5. However, according to S2, the required annual average water demand of the marshes is 11 × 10^9^ m^3^/year (Iraqi Ministries of Environment et al. 2006). This amount constitutes 14% and 21% of the present and future total annual average inflow of the Tigris and Euphrates Rivers. Accordingly, the expected deficit and net available water for the considered marshes by the years 2025 and 2035 will be as listed in Table 6. Hence, the expected deficit in net available water for the Hawizeh, Hammar, and Central Marshes by the years 2025 (2035) for S1 will be approximately 37% (47%), 14% (18%), and 24% (30%), respectively. However, for S2, it will be almost equal by the 2 years 2025 and 2035 with 11% for the Hawizeh, 4% for the Hammar, and 11% for the Central Marshes.

**Table 5:** Expected deficit and net available water for the years 2025 and 2035, according to S1

Marsh	Restoration percentage (%)	Main feeding sources	Deficit (10^9^ m^3^/year)		Required (10^9^ m^3^/year)	Available (10^9^ m^3^/year)	
			2025	2035		2025	2035
Hawizeh	65	Tigris	0.81	1.03	2.00	1.19	0.97
	75				2.40	1.59	1.37
Hammar	55	Euphrates	0.62	0.79	4.00	3.38	3.21
	75				5.00	4.38	4.21
Central	50	Tigris	0.81	1.03	3.25	2.44	2.22
	75				3.60	2.79	2.57

**Table 6:** Expected deficit and net available water for the years 2025 and 2035, according to S2

Marsh	Restoration percentage (%)	Main feeding sources	Deficit (%)		Required (10^9^ m^3^/year)	Available (10^9^ m^3^/year)	
			2025	2035		2025	2035
Hawizeh	65	Tigris	10.7	11.25	2.00	1.79	1.78
	75				2.40	2.14	2.13
Hammar	55	Euphrates	4.1	4.28	4.00	3.84	3.78
	75				5.00	4.80	4.78
Central	50	Tigris	10.7	11.25	3.25	2.90	2.88
	75				3.60	3.21	3.20

### Expected decrease in the restorable areas of Iraq marshlands

**Fig. 14:**
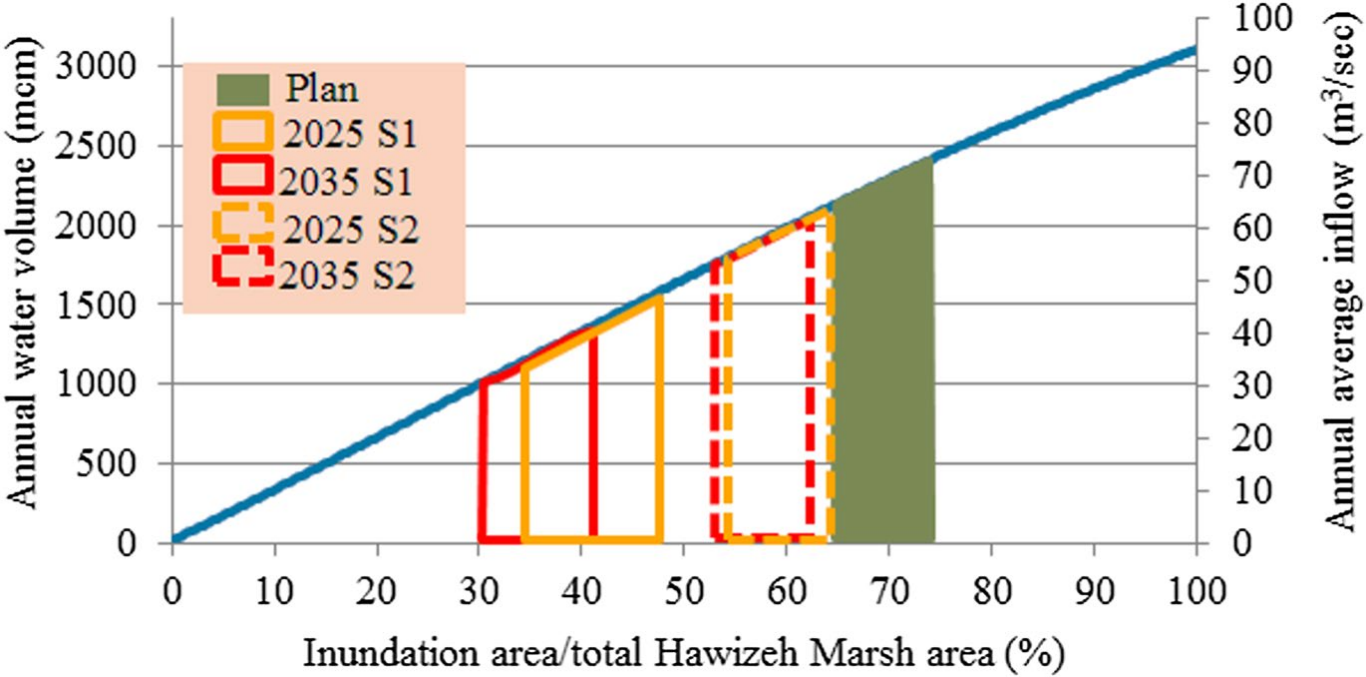
Possible inundation area of Hawizeh Marsh by the years 2025 and 2035 for S1 and S2

**Fig. 15:**
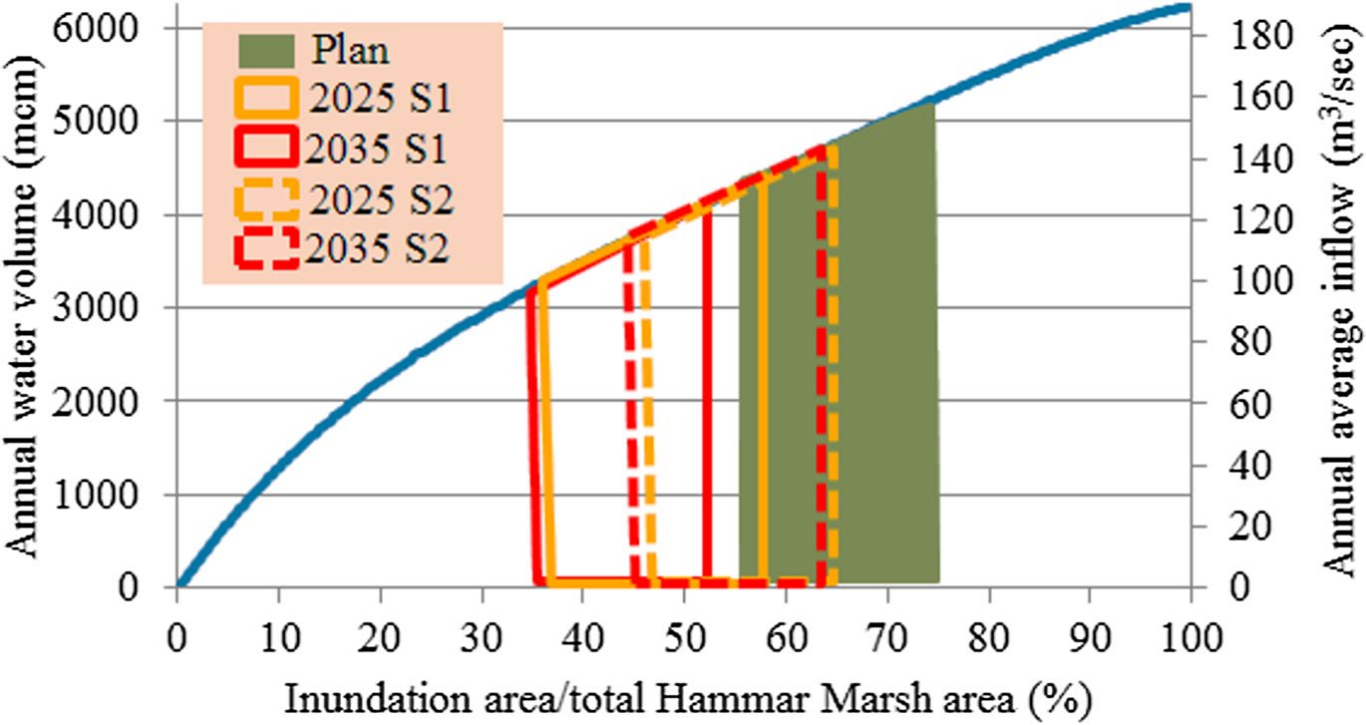
Possible inundation area of Hammar Marsh by the years 2025 and 2035 for S1 and S2

**Fig. 16:**
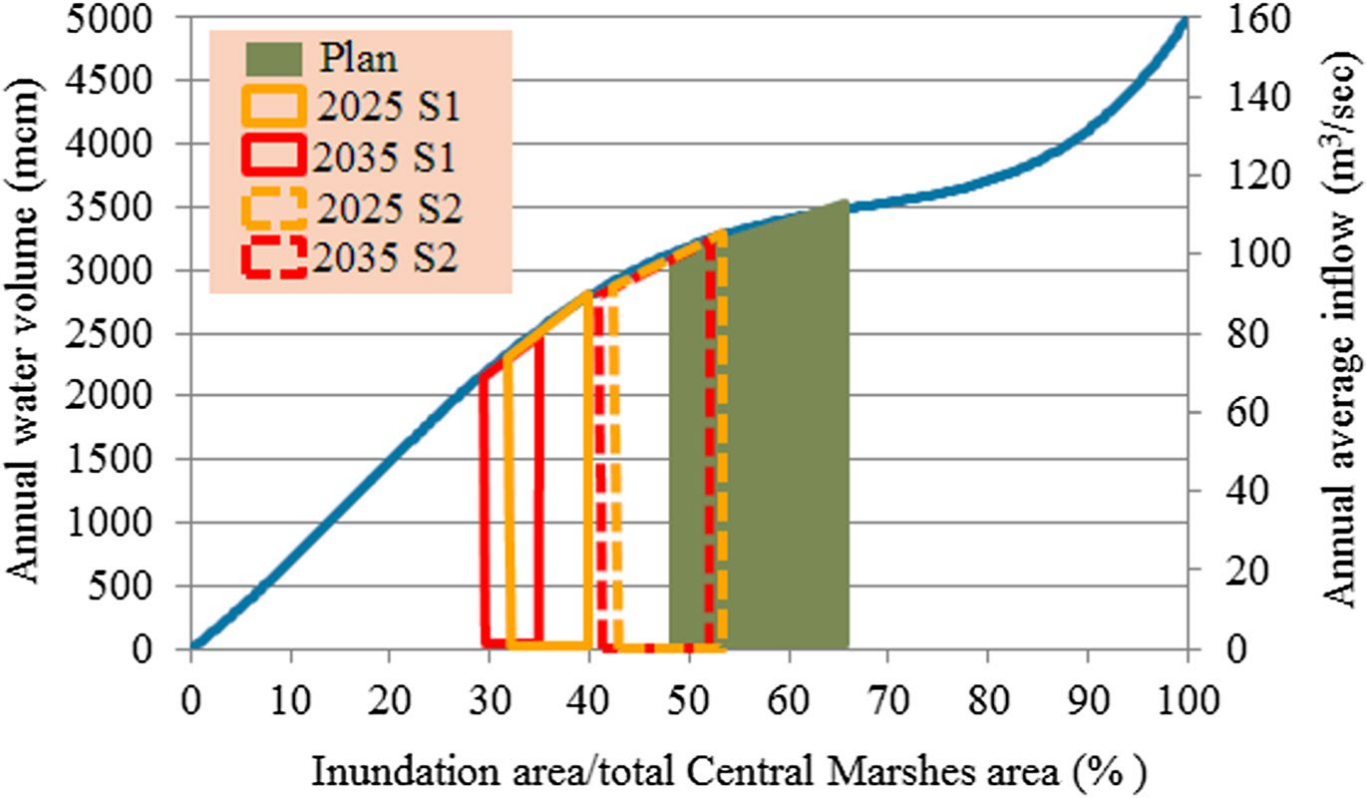
Possible inundation area of the Central Marshes by the years 2025 and 2035 for S1 and S2

Based on the estimated deficit and available water, shown in **Tables** **5** and **6**, and according to the relationship between the available annual water volume and percentage of inundation area to total marsh area for the three marshes (Figs. 4, 5, and 6), the percentage of areas possible to be inundated by the years 2025 and 2035 according to S1 and S2 for restoration 75% (65%) of the total area of Hawizeh Marsh will be 48% and 35% (42% and 30%), for restoration 75% (55%) of Hammar marsh will be 58% and 38% (53% and 35%), and for restoration 75% and 50% of the Central Marshes will be 40% and 33% (37% and 29%), as shown in Figs. 14, 15, and 16. Consequently, according to the S1 (S2), Hawizeh, Hammar, and the Central Marshes are expected to lose approximately 25.0% (37.5%), 9.5% (28.5%), and 24.0% (31.5%) of their eligible restoration areas (Hawizeh Marsh (1377 km^2^), Hammar Marsh (1762 km^2^), and the Central Marshes (2420 km^2^), respectively.

## Conclusions

Evaluation of the recorded field data and results of the land cover classification through implementing the satellite image–based index model showed that the re-inundation areas (Re) are usually greater than the vegetation cover (VC) areas with an average (maximum) value of 13.5% (21.1%), 26.0% (46.0%), and 0.3% (35.1%) for Hawizeh, Hammar, and the Central Marshes, respectively. For the three marshes, the maximum Re (VC) was in the year 2014 (2018). The relative stability of inundation rates during the period 2014–2018 contributed to the increase in VC during this period compared to the period before 2014. Also, the classification of the marshes’ land cover showed that there was a slight increase in the dense and medium VCs in Hawizeh Marsh, whereas enhancement in these classes was better in Hammar Marsh. However, in the Central Marshes, although the situation after 2014 was slightly better than the years before, the situation fluctuated and there was no noticeable improvement in the areas of dense or medium VCs. Furthermore, despite the maximum Re of the Hawizeh, Hammar, and Central Marshes being 81%, 91%, and 67%, respectively, which satisfy the targeted levels in the restoration plan, this high rate was maintained for just 1 year (2014). However, the application of the most optimistic scenarios that are less harmful to the marshes shows that the Hawizeh, Hammar, and Central Marshes are expected to lose approximately 25.0–37.5%, 9.5–28.5%, and 24.0–31.5% of their eligible restoration areas, respectively. The findings of the VC investigation highlight the necessity of maintaining consistent and stable discharge levels in the marshes to ensure the sustainability of vegetation cover. Also, limited water resources and inconsistent inundation rates have impeded the successful implementation of restoration plans for sustaining these marsh ecosystems. The restoration of Iraqi marshes is threatened due to the anticipated severe water scarcity and the absence of alternative solutions for maintaining necessary water resources. While sufficient water supplies might be available to inundate vast marshland areas during certain wet years, this is not consistent enough to guarantee long-term ecosystem stability. Consequently, decision-makers face the challenge of identifying suitable alternatives for securing additional water resources, such as recycling the drainage and sewage water and/or recourse to reducing the areas targeted by inundation, and being satisfied with the areas that can be sustainable and maintaining the current status of the rest of the regions as an emerging ecosystem characterized by lands that are inundated every few years depending on the frequency of wet years. This ecosystem can be a habitat for new species of plants, animals, and other organisms. Accordingly, steps must be urged to develop plans and programs to maintain the sustainability of these emerging ecosystems within the frameworks of climate change and the conditions of scarcity of water resources and water and air pollution to ensure that they are not lost in the future.

## Data Availability

The data generated or analyzed during this study are available from the corresponding author upon reasonable request.
